# DLL3 Expression in Neuroendocrine Carcinomas and Neuroendocrine Tumours: Insights From a Multicentric Cohort of 1294 Pulmonary and Extrapulmonary Neuroendocrine Neoplasms

**DOI:** 10.1007/s12022-025-09854-3

**Published:** 2025-03-28

**Authors:** Maxime Schmitt, Hanibal Bohnenberger, Detlef Klaus Bartsch, Daniel-Christoph Wagner, Anne-Sophie Litmeyer, Albert Grass, Anja Rinke, Christine Koch, Marcus Kremer, Matthias Evert, Bruno Märkl, Alexander Quaas, Markus Eckstein, Konrad Steinestel, Carsten Denkert, Katja Steiger, Günter Klöppel, Atsuko Kasajima, Markus Tschurtschenthaler, Sebastian Foersch, Moritz Jesinghaus

**Affiliations:** 1 Institute of Pathology, Philipps University Marburg and University Hospital Marburg, Baldingerstrasse, Marburg Germany; 2 Institute of Pathology, University Hospital Göttingen, Göttingen, Germany; 3 Department of Visceral, Thoracic- and Vascular-Surgery, Philipps University Marburg and University Hospital Marburg, Marburg, Germany; 4 Institute of Pathology, University Hospital Mainz, Mainz, Germany; 5 Department of Gastroenterology, Endocrinology and Infectious Diseases, Philipps University Marburg and University Hospital Marburg, Marburg, Germany; 6 Department of Internal Medicine I, University Hospital Frankfurt, Frankfurt, Germany; 7 Institute of Pathology, Städtisches Klinikum München, Munich, Germany; 8 Institute of Pathology, University of Regensburg, Regensburg, Germany; 9 Institute of Pathology, University Hospital Augsburg, Augsburg, Germany; 10 Institute of Pathology, University Hospital Cologne, Cologne, Germany; 11 Institute of Pathology, University Hospital Erlangen, Friedrich-Alexander-University Erlangen-Nuremberg, Erlangen, Germany; 12 Institute of Pathology and Molecular Pathology, Department XIII, Bundeswehrkrankenhaus Ulm, Ulm, Germany; 13 Department of Pathology, School of Medicine and Health, Technical University Munich, Munich, Germany; 14 Institute for Translational Cancer Research (TranslaTUM), School of Medicine, Technical University of Munich, Munich, Germany; 15 Institute of Experimental Cancer Therapy, School of Medicine, Technical University of Munich, Munich, Germany; 16 Division of Translational Cancer Research, German Cancer Research Center (DKFZ) and German Cancer Consortium (DKTK), Heidelberg, Germany

**Keywords:** DLL3, Neuroendocrine neoplasms, Neuroendocrine tumour, Neuroendocrine carcinoma

## Abstract

Delta-like ligand 3 (DLL3) is frequently expressed in pulmonary small cell neuroendocrine carcinoma (SCNEC) and has emerged as a promising therapeutic target. However, limited data on DLL3 expression in other neuroendocrine neoplasms (NEN), such as extrapulmonary SCNEC, large cell neuroendocrine carcinomas (LCNEC), mixed neuroendocrine-non-neuroendocrine neoplasms (MiNEN), gastroenteropancreatic neuroendocrine tumours (GEP-NET), and pulmonary carcinoids, impedes an estimation if other types of NEN might be suitable candidates for anti-DLL3 therapies. We evaluated DLL3 expression in 1294 NEN and 479 non-neuroendocrine carcinomas, correlating the findings with histological subtypes, tumour localisation, and overall survival (OS). Furthermore, we explored the concordance of DLL3 expression during metastatic progression in 67 paired primary NEN and metastases. DLL3 expression was significantly higher in NEC (64.0%) compared to GEP-NET and pulmonary carcinoids (10.1%, *p* < 0.001), particularly in SCNEC (80.4%), followed by LCNEC (62.6%) and MiNEN (28.6%). DLL3 was common in pulmonary carcinoids (41.5%), but rare in GEP-NET (5.1%) and non-neuroendocrine carcinomas (1.3%). Overall DLL3 expression was highly concordant between metastases and corresponding primary NEN (92.5%, *p* < 0.001). In univariable analyses, DLL3-expressing pulmonary carcinoids (*p* = 0.005) and GEP-NET (*p* = 0.018) were associated with decreased OS, but this was not retained in multivariable analyses adjusting for stage and grade (*p* = n. s.). No prognostic impact was observed in pulmonary (*p* = 0.708) or GEP-NEC (*p* = 0.87). Our study highlights significant differences in DLL3 expression across NEN subtypes and localisations, with largely concordant expression in metastases. DLL3-based therapies may be effective in many NEC and pulmonary carcinoids, while DLL3 appears to be a minor therapeutic target for GEP-NET and non-neuroendocrine carcinomas.

**Supplementary Information:**

The online version contains supplementary material available at 10.1007/s12022-025-09854-3.

## Introduction

Epithelial neuroendocrine neoplasms (NEN) encompass well-differentiated neuroendocrine tumours (NET) and poorly differentiated neuroendocrine carcinomas (NEC). Despite their metastatic potential, NET frequently show long-term clinical courses and have a comparably favourable prognosis. In contrast, NEC are highly aggressive neoplasms characterized by a markedly inferior prognosis compared to NET but also to non-neuroendocrine carcinomas arising from the same primary site [1–6]. Although being distinguished by their genetic signatures and histomorphology, both NET and NEC are characterized by their ability to originate as primary lesions in many anatomical locations throughout the body. Moreover, both entities often present in advanced clinical stages at the time of diagnosis, underscoring the necessity for enhancements in diagnostic and therapeutic strategies [1–3].

Delta-like ligand 3 (DLL3), which functions as an inhibitory ligand within the Notch pathway [7], has emerged as a promising target for the treatment of NEN, especially for small cell NEC (SCNEC) of the lung [8–12]. Various therapy-modalities for pulmonary SCNEC that target DLL3—such as T-cell engager molecules, antibody–drug conjugates and CAR T-cells—are currently undergoing clinical trials [7, 8]. Recently, Tarlatamab, a bispecific T-cell engager targeting DLL3 [13–15], has been granted accelerated approval by the Food and Drug Administration for extensive pulmonary SCNEC with disease progression on or after platinum-based chemotherapy. Furthermore, recent studies have demonstrated the principal feasibility of DLL3-PET CT imaging for SCNEC in preclinical murine models [16], but also in patients [17]. These data, combined with the described absence of DLL3 expression in non-neoplastic tissues [16], argue that DLL3 may not only be a protein of interest for the diagnostics and treatment of pulmonary SCNEC, but perhaps also for NEN in general. However, in contrast to pulmonary SCNEC, data regarding DLL3 expression in extrapulmonary SCNEC, pulmonary and extrapulmonary large cell NEC (LCNEC), mixed neuroendocrine-non-neuroendocrine neoplasms (MiNEN), gastroenteropancreatic NET (GEP-NET), and pulmonary carcinoids is scarce [8, 18]. This precludes a comprehensive overview of the general DLL3 expression landscape in NEN and therefore also an estimation regarding which types of NEN might be considered viable targets for anti-DLL3 therapies or imaging modalities.

Our study aimed to determine whether DLL3 expression is generally exclusive to NEC or if similar expression levels are also present in NET or non-neuroendocrine carcinomas. Furthermore, we investigated the distribution of DLL3 expression among different subtypes and grades of NEC and NET, and its association with the localization of the primary tumour. Finally, we explored whether DLL3 expression could identify distinct prognostic subgroups within NET or NEC. To address these questions, we analysed DLL3 expression in a large multicentric cohort of 1294 NET and NEC (301 pulmonary, 993 extrapulmonary), along with 67 matched NEN metastases and 479 non-neuroendocrine carcinomas.

## Material and Methods

### Multicentric NEN Cohort

We established a multicentric cohort of 1294 primary NEN from 1143 patients. FFPE tissue blocks of the tumours were retrieved from the archives of the pathology departments of the University Hospital Marburg, the University Hospital rechts der Isar of the Technical University of Munich, the Munich Municipal Hospital, the University Hospital Mainz, the University Hospital Cologne, the University of Regensburg, the University Hospital Erlangen-Nuremberg, the University Hospital Göttingen, and the University Hospital Augsburg. The median age at diagnosis was 61 years, 485 patients were females (42.4%) and 659 (57.6%) were males. Survival and clinicopathological data were collected from local cancer registries or hospital records. Overall survival (OS) information was available for 578 patients (44.7%), based on recorded deaths from any cause.

All tumours were diagnosed according to the respective WHO classifications of their organ system and all cases were morphologically reviewed prior to inclusion in this study [1–3]. A neoplasm was classified as a NET, if it showed a well-differentiated neuroendocrine morphology, including monomorphic nuclei with granular chromatin, and organoid architecture, along with strong expression of neuroendocrine markers like synaptophysin or chromogranin A (except for rectal NET) [19] accompanied by expression of cytokeratins. NET were graded according to their Ki-67 proliferation index (G1: < 3%, G2: 3–20%, G3: > 20%). The respective organ-specific criteria were applied for the diagnosis of pulmonary typical carcinoids (TC) and atypical carcinoids (AC) [2]. Pulmonary and extrapulmonary NEC were also diagnosed according to the criteria given by the respective WHO classifications [1–3]. Poorly differentiated cancers with a high mitotic index and/or necrosis with an architecture showing solid sheets of medium to large-sized tumour cells with rounded vesicular nuclei exhibiting prominent nucleoli were classified as large cell NEC (LCNEC). The diagnosis of a small cell NEC (SCNEC) was made for poorly differentiated neoplasms with a high mitotic activity consisting of small to medium-sized cells with scant basophilic cytoplasm and elongated hyperchromatic nuclei lacking distinctive nucleoli. A MiNEN was diagnosed when a morphologically distinct neuroendocrine neoplasm was mixed with a non-neuroendocrine carcinoma component, with each component comprising more than 30% of the entire tumour. Our cohort included only the combination of a NEC with a non-neuroendocrine carcinoma; the combination of a NET or AC/TC with a non-neuroendocrine carcinoma was not present in our cohort.

Using patient tissue from Marburg, Göttingen, Cologne, Munich, Mainz, Regensburg, Augsburg, and Erlangen, we assembled a Tissue microarray (TMA) comprising up to four tumour cores per case using the TMA grand master system (Sysmex/3DHistech, Budapest, Hungary). Tumours with insufficient fixation or insufficient tumour material on the TMA were excluded from this study.

### Non-neuroendocrine Carcinoma Cohort

Furthermore, we investigated a second cohort of 479 non-neuroendocrine carcinomas from the University Hospital rechts der Isar of the Technical University of Munich and the University Hospital Marburg, consisting of 239 primary resected colorectal adenocarcinomas, 91 pancreatic adenocarcinomas, 69 gastric adenocarcinomas, and 76 pulmonary carcinomas (58 pulmonary adenocarcinomas, 18 pulmonary squamous cell carcinomas), all lacking morphological and immunohistochemical criteria of neuroendocrine differentiation as described above. All cases were assembled on TMA with two separate tissue cores per patient, which were investigated according to their expression of DLL3 in a similar fashion as the NEN, as described in the following paragraph.

### NEN Metastases

To exploratorily assess DLL3 expression variability between primary tumours and metastases, we analysed whole tissue slides from 67 metastatic lesions derived from 52 patients/primary tumour sites, randomly selected from the NEN cohort. DLL3 expression was evaluated as described in the following paragraph.

### Immunohistochemical Analyses of DLL3

TMA comprising tissue cores from 1227 NEN and 479 non-neuroendocrine carcinomas were stained on a BenchMARK XT/LT stainer with a DLL3 antibody (VENTANA DLL3 SP347 Assay, ready to use) visualized by an OptiView DAB IHC Detection Kit (Roche Diagnostics, Mannheim, Germany). Furthermore, 67 whole slides of patients with pulmonary SCLC, LCNEC, TC, and AC (biopsies and resections) were included. DLL3 expression was evaluated manually by an experienced pathologist (MJ). Only a clear membranous and/or cytoplasmic staining of DLL3 was considered specific [20]. For each individual patient, the cumulative percentage of positive cells (range: 0–100%) for all cores was assessed. DLL3 expression intensity was graded as strong (promptly visible in scanning magnification), moderate (clearly visible staining but notably weaker), weak (barely perceptible and only notable in high magnifications), and negative (no staining reaction). Next, all NEN were assigned to different DLL3 expression groups according to the immunoreactive score (IRS) [21], which was calculated by multiplying the maximum-staining-intensity-score (ranging from 0 to 3) with the percentage-of-expressing-cells-score (ranging from 0 to 4). Subsequently, we assigned four DLL3 expression groups based on these scores: DLL3 negative (IRS 0–1), DLL3 low (IRS 2–3), DLL3 moderate (IRS 4–8), and DLL3 strong (IRS 9–12) [22, 23]; any IRS score ≥ 2 was considered DLL3 positive. Table 1 shows the algorithm to determine the IRS as well as the resulting DLL3 expression groups in detail.

**Table 1:** Algorithm to determine DLL3 expression scores according to the IRS score

Intensity score	Staining intensity	Percentage score	Percentage of positive cells
0	No staining reaction	0	0%
1	Weak staining reaction	1	< 10%
2	Moderate staining reaction	2	10–50%
3	Strong staining reaction	3	51–80%
		4	> 80%
**IRS = score (staining intensity) × score (percentage of positive cells)**			
**DLL3 expression groups **			
IRS 0–1		DLL3 negative	
IRS 2–3		DLL3 weak	
IRS 4–8		DLL3 moderate	
IRS 9–12		DLL3 strong	

To assess intercomponent heterogeneity in detail in MiNEN, we conducted a separate analysis of whole-slides from 10 cases that displayed a mosaic-like arrangement between the NEC and non-neuroendocrine components, with both morphological components distinctly separated.

In order to test interobserver reliability, DLL3 expression was independently investigated in 160 randomly selected NET and NEC by an additional observer (MS) reaching an almost perfect interobserver concordance for the IRS groups (Cohen’s kappa *κ* = 0.88, *p* < 0.001). Discrepant cases were discussed until a consensus was reached. In addition, the DLL3-staining reaction on one TMA was compared to their corresponding whole slides, reaching a substantial concordant staining when the distribution between the exact IRS groups was investigated (Cohen’s kappa *κ* = 0.79; *p* < 0.001) and an almost perfect concordance when only simplified DLL3 expression groups (DLL3-negative vs. DLL3-positive) were considered (Cohen’s kappa *κ* = 0.90; *p* < 0.001).

### Statistics

Associations between morphological characteristics and clinicopathological parameters were tested using the chi-square (*χ*^2^) test and Fisher’s exact test (two-sided). Univariable survival probabilities were estimated with the Kaplan–Meier method, and differences in survival were assessed using log-rank tests. Mean and median survival times are reported with 95% confidence intervals (CI). The hazard ratio (HR) for univariable survival analyses was determined using the univariate Cox proportional hazards regression model. Interobserver agreement was measured using Cohen’s kappa and interpreted according to Landis et al. (*κ* < 0: less than chance agreement, *κ* = 0.01–0.20: slight agreement, *κ* = 0.21–0.40: fair agreement, *κ* = 0.41–0.60: moderate agreement, *κ* = 0.61–0.80: substantial agreement, *κ* = 0.81–0.99: almost perfect agreement) [24]. Exploratory *p*-values of ≤ 0.05 were considered statistically significant. All analyses were conducted using IBM SPSS Statistics Version 28.0 (IBM Corp, Armonk, NY, USA).

## Results

### Clinicopathological Features of the NEN Cohort

**Fig. 1:**
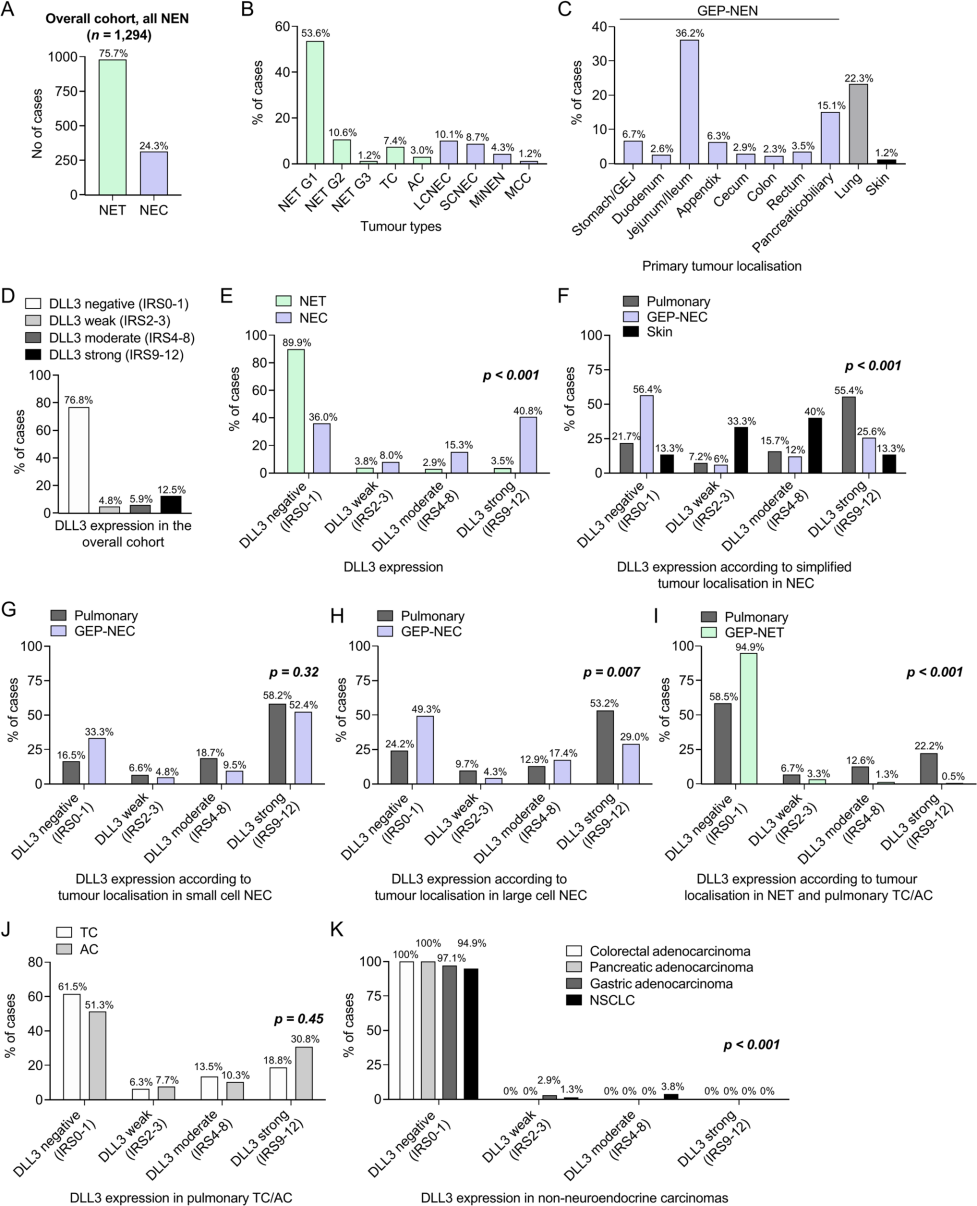
Overview of the cohort and DLL3 expression in different NEN. **A** Frequency of NET (including AC/TC) and NEC (including MiNEN) in the overall cohort of 1294 NEN. **B** Frequency of different NET (G1/G2/G3, TC/AC) and NEC (SCNEC/LCNEC/MiNEN) subtypes in the overall cohort. **C** Detailed localisations of the investigated NEN in the overall cohort. **D** Frequency of DLL3 expression groups according to the IRS in the overall cohort (all NEN). **E** General comparison of DLL3 expression groups according to the IRS between NET and NEC. **F** Frequency of DLL3 expression groups according to the IRS in NEC between different simplified localisations (pulmonary, GEP-NEC, skin). **G** Frequency of DLL3 expression groups according to the IRS in pulmonary and gastroenteropancreatic SCNEC. **H** Frequency of DLL3 expression groups according to the IRS in pulmonary and gastroenteropancreatic LCNEC. **I** Frequency of DLL3 expression groups according to the IRS in gastroenteropancreatic NET and pulmonary TC/AC. **J** Frequency of DLL3 expression groups according to the IRS in pulmonary TC and AC. **K** Frequency of DLL3 expression groups according to the IRS in pulmonary and gastroenteropancreatic non-neuroendocrine carcinomas. DLL3, Delta-like-protein 3; NEN, neuroendocrine neoplasm; NET, neuroendocrine tumour; NEC, neuroendocrine carcinoma, LCNEC, large cell neuroendocrine carcinoma; SCNEC, small cell neuroendocrine carcinoma; MiNEN, mixed neuroendocrine-non-neuroendocrine carcinoma; TC, typical carcinoid; AC, atypical carcinoid; GEP, gastroenteropancreatic; MCC, Merkel cell carcinoma

Our multicentric NEN cohort included 980 primary NET (75.7%, including pulmonary carcinoids) and 314 primary NEC (24.3%, Fig. 1A) and comprised 301 (23.3%) pulmonary, 978 (75.6%) gastroenteropancreatic NEN (GEP-NEN) and 15 (1.2%) cutaneous NEN (Merkel cell carcinomas, detailed localisation see Fig. 1C). Besides 803 (62.1%) unifocal NET from various sites, our cohort included 177 (13.7%) jejunoileal NET from 27 individual patients with multifocal jejunoileal NET [25]. Of the 314 NEC (166, 12.8% pulmonary/133, 10.3% GEP-NEC/15, 1.2% cutaneous), 131 (41.7%) were diagnosed as LCNEC, 112 (35.7%) were diagnosed as SCNEC and 56 (17.8%) were MiNEN along with 15 (4.8%) Merkel cell carcinomas. Among well-differentiated NEN, 693 (70.7%) NET were graded as G1, 137 (14.0%) as G2, and 15 (1.5%) as G3, in addition to 96 (9.8%) TC and 39 (4.0%) AC of the lung.

The additional NEN metastases cohort comprised metastases of 51 (76.1%) metastasised NET and 16 (23.9%) NEC. Regarding metastatic localisation, 30 (44.8%) metastases were located in the liver, 32 (47.8%) in lymph nodes and five (7.5%) at other metastatic sides (peritoneum, soft tissue, adrenal gland) (for details regarding the cohort, see Supplementary Table 2).

### DLL3 Expression in the Overall NEN Cohort

**Fig. 2:**
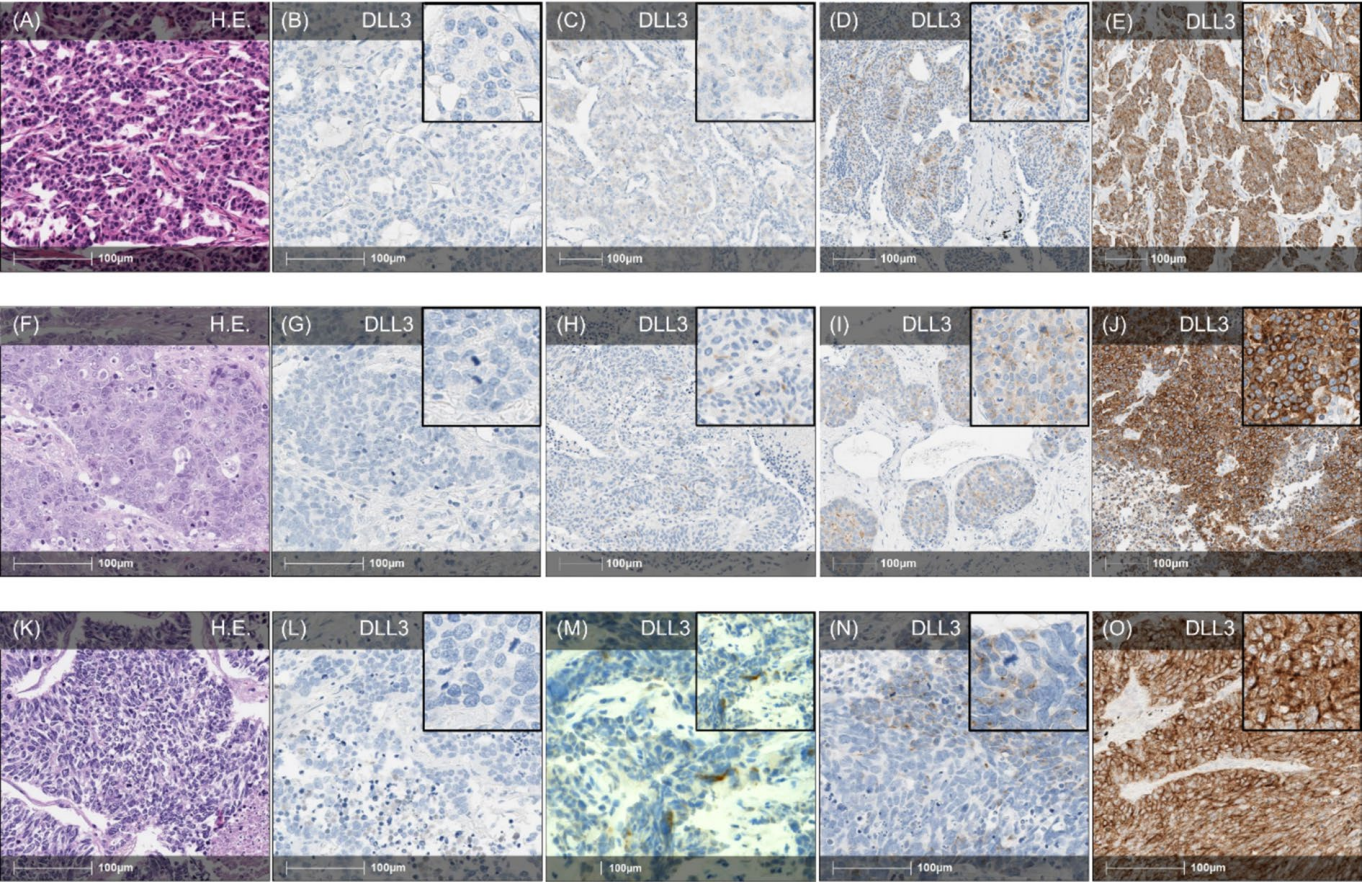
Expression of DLL3 in pulmonary NEN. **A**–**E** Pulmonary carcinoids (HE, **A**). **B** Example of pulmonary typical carcinoid with complete absence of DLL3 expression (IRS 0). **C** Example of weak DLL3 expression in pulmonary typical carcinoid showing a weak expression intensity in > 10% of tumour cells (IRS 2). **D** Moderate DLL3 expression in pulmonary typical carcinoid with up to strong cytoplasmatic staining intensity, which is restricted to > 10% of tumour cells (IRS 6). **E** Strong DLL3-expression in pulmonary atypical carcinoid with strong expression intensity in > 80% of carcinoid cells (IRS 12). **F**–**J** Examples of different pulmonary LCNEC (HE, **F**). **G** Example of negative DLL3 expression in pulmonary LCNEC without any cytoplasmatic DLL3 expression (IRS 0). **H** Pulmonary LCNEC with weak DLL3-expression demonstrating an up to strong staining intensity in < 10% of carcinoma cells (IRS 3). **I** Moderate DLL3 expression in pulmonary LCNEC with strong staining intensity in > 10% of tumour cells (IRS 6). **J** Example of pulmonary LCNEC with strong cytoplasmatic staining reaction in > 80% of carcinoma cells falling into strong DLL3 expression group (IRS 12). **K**–**O** Different pulmonary SCNEC (**K**, HE). **L** Pulmonary SCNEC with no expression of DLL3 at all (IRS 0). **M** Example of weak DLL3 expression demonstrating up to strong staining reaction in < 10% of tumour cells (IRS 3). **N** Moderate DLL3 expression in pulmonary SCNEC with up to strong cytoplasmatic staining reaction in > 10% of tumour cells (IRS 6). **O** Example of pulmonary SCNEC with strong staining reaction in almost all carcinoma cells meaning an overall strong DLL3 expression (IRS 12). Overview: × 20 magnification, Inlay: × 100 magnification. HE, hematoxylin and eosin; DLL3, Delta-like-protein 3; IRS, immunoreactive score; NEN, neuroendocrine neoplasm; LCNEC, large-cell neuroendocrine carcinoma; SCNEC, small-cell neuroendocrine carcinoma

**Fig. 3:**
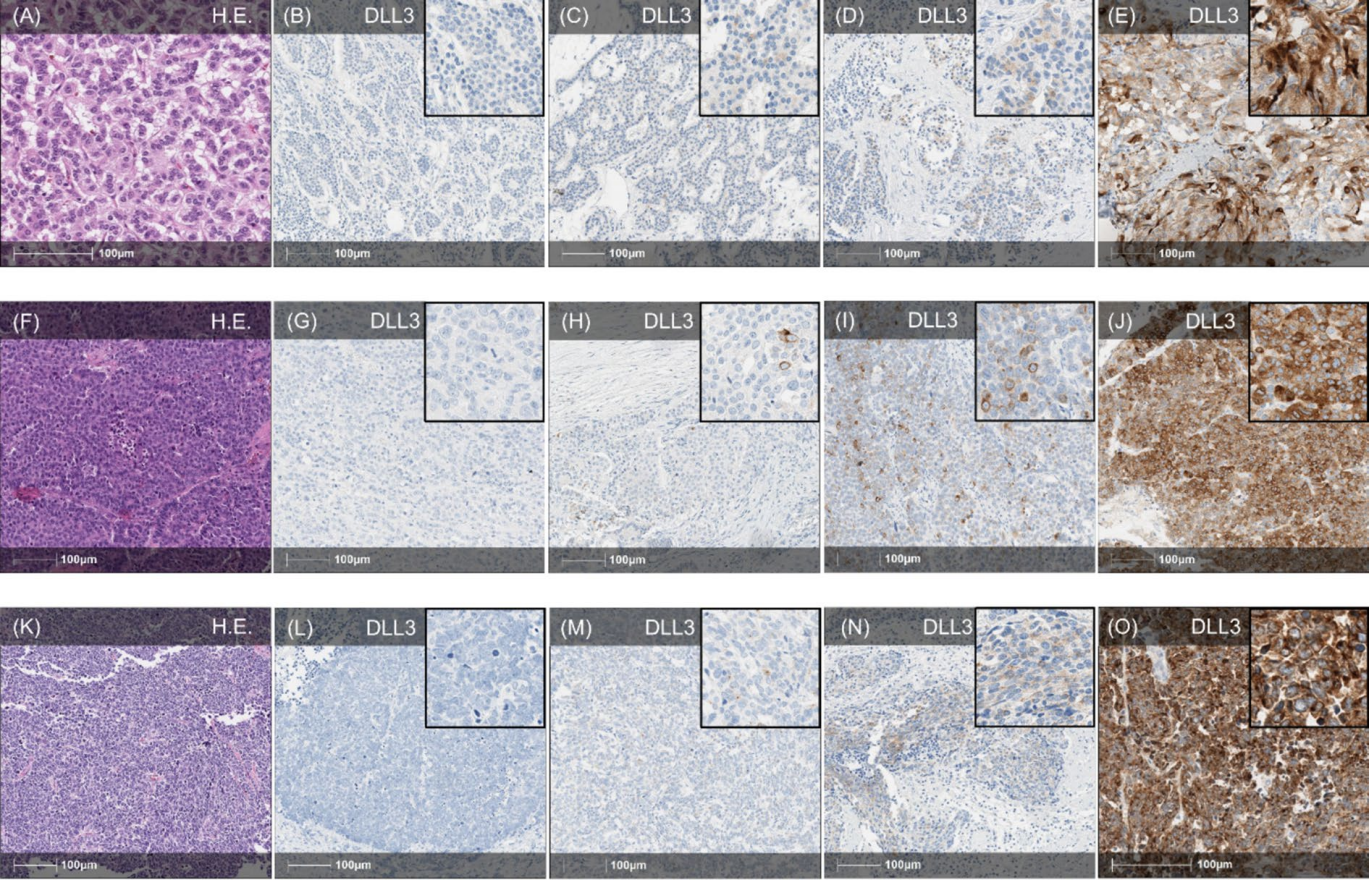
DLL3 expression in gastroenteropancreatic NEN. **A**–**E** Different pancreatic NET (HE, A). **B** Example of pancreatic NET with complete absence of DLL3 expression (IRS 0). **C** Weak DLL3 expression in pancreatic NET with an up to moderate cytoplasmatic staining reaction in < 10% of tumour cells (IRS 2). **D** Example of pancreatic NET with moderate expression intensity in > 10% of tumour cells meaning a moderate DLL3 expression (IRS 4). **E** Strong DLL3 expression in pancreatic NET with strong staining intensity in > 50% of tumour cells (IRS 9). **F**–**J** Examples of DLL3 expression in different GEP-LCNEC (HE, **F**). **G** Example of pancreatic LCNEC with complete absence of DLL3 expression (IRS 0). **H** Example of weak DLL3 expression in rectal LCNEC with an up to strong expression intensity in < 10% of carcinoma cells (IRS 3). **I** Moderate DLL3 expression in rectal LCNEC demonstrating an up to strong staining intensity in > 10% of tumour cells (IRS 6). **J** Example of pancreatic LCNEC with strong cytoplasmatic staining reaction in almost all tumour cells meaning a strong DLL3 expression (IRS 12). **K**–**O** SCNEC of different sides of the GEP (**K**, HE). **L** Example of SCNEC in colon with no expression of DLL3 at all (IRS 0). **M** SCNEC of the colon demonstrating a weak staining reaction in > 10% of tumour cells corresponding to an overall weak DLL3 expression (IRS 2). **N** Moderate DLL3 expression in rectal SCNEC showing a moderate staining reaction in > 50% of carcinoma cells (IRS 6). **O** Example of gastric SCNEC with strong DLL3 expression in almost all tumour cells (IRS 12). Overview: × 20 magnification, Inlay: × 100 magnification. HE, hematoxylin and eosin; DLL3, Delta-like-protein 3; IRS, immunoreactive score; NEN, neuroendocrine neoplasm; LCNEC, large-cell neuroendocrine carcinoma; SCNEC, small-cell neuroendocrine carcinoma; GEP, gastroenteropancreatic

Expression of DLL3 (any degree) was observed in 300/1294 (23.2%) NEN, while 994 (76.8%) neoplasms were DLL3 negative (Fig. 1D). According to the IRS, 62 (4.8%) neoplasms showed a weak (IRS 2–3), 76 (5.9%) showed a moderate (IRS 4–8) and 162 (12.5%) showed a strong expression (IRS 9–12). Examples of the different DLL3 expression groups according to the IRS are given in Fig. 2 for pulmonary NEN and in Fig. 3 for GEP-NEN.

### DLL3 Expression in NEC

DLL3 was expressed in 201/314 (64.0%) NEC. SCNEC (90/112, 80.4%) and Merkel cell carcinoma (13/15, 86.7%) showed a significantly higher frequency of DLL3 expression (any degree; *p* < 0.001) compared to LCNEC (82/131, 62.6%) and MiNEN (16/56, 28.6%). In terms of expression intensity, the rate of a strong DLL3 expression was significantly pronounced in SCNEC (64/112, 57.1%; *p* < 0.001) compared to LCNEC. In MiNEN, DLL3 expression was predominantly concordant across components when all expression intensities were considered, with concordance observed in 90% of the cases. Notably, when DLL3 positivity was present, the non-neuroendocrine component consistently displayed weaker expression intensity than the NEC component (Supplementary Fig. 1).

Pulmonary NEC (130/166, 78.3%; *p* < 0.001) showed a significantly increased rate of general DLL3 expression compared to GEP-NEC (58/133, 43.6%; *p* < 0.001), and also, the rate of strongly positive cases (pulmonary NEC: 92/166, 55.4% vs. GEP-NEC: 34/133, 25.6%; *p* < 0.001) was significantly enriched in pulmonary NEC (Fig. 1F). When we analysed pulmonary SCNEC vs. extrapulmonary SCNEC separately, we observed no significant differences (*p* = 0.32) in DLL3 expression (Fig. 1G). In contrast, pulmonary LCNEC (*p* = 0.007) and MiNEN (*p* = 0.04) were significantly more often DLL3 positive compared to their extrapulmonary counterparts (Fig. 1H). For detailed distribution of DLL3 expression groups in NEC/MiNEN according to their detailed anatomic sides also see Supplementary Fig. 2A.

### DLL3 Expression in NET and Pulmonary TC/AC

DLL3 was expressed in 99/980 (10.1%) NET/TC/AC, with 37 (3.8%) showing a weak, 28 (2.9%) showing a moderate and 34 (3.5%) displaying a strong expression, while 881 (89.9%) neoplasms were completely DLL3 negative.

Regarding tumour localisation, pulmonary AC (19/39, 48.7%) and TC (37/96, 38.5%) showed a much higher rate of DLL3 positivity compared to GEP-NET (43/845, 5.1%; *p* < 0.001). While the majority of DLL3 positive pulmonary carcinoids (DLL3 positive TC and AC: 56/135, 41.5%) showed a strong (30/135; 22.2%) or moderate (17/135; 12.6%) expression (*p* < 0.001), the majority of DLL3 positive GEP-NET showed a weak expression (28/845, 3.3%) (Fig. 1I–J, Supplementary Fig. 2B, Supplementary Table 1).

In pulmonary carcinoids, we did not observe significant differences in general DLL3 expression (*p* = 0.28) or the distribution of DLL3 expression groups (*p* = 0.45) between TC and AC (Fig. 1J). In GEP-NET, NET G3 (8/15, 53.3%) showed a much higher fraction of DLL3 expression compared to NET G2 (6/15, 4.4%) and NET G1 (29/693, 4.2%; *p* < 0.001). For detailed distribution of DLL3 expression groups in NET/TC/AC according to their anatomic sides, also see Supplementary Fig. 2B and Supplementary Table 1.

### DLL3 Expression in NET Vs. NEC

In the overall cohort, general DLL3 expression (any degree) was far more frequent in NEC (201/314; 64.0%; *p* < 0.001) than in NET and pulmonary carcinoids (99/980; 10.1%), also with much higher frequencies of moderate and strongly positive NEC (NEC: 176/314, 56.1%; NET/TC/AC: 62/980, 6.3%; *p* < 0.001) (Fig. 1E).

In separate analyses of both pulmonary and GEP-NEN, DLL3 expression was again strongly associated with NEC (*p* < 0.001, respectively) with higher fractions of DLL3 expression in pulmonary NEC (130/166, 78.3%) compared to GEP-NEC (58/133, 43.6%).

The comparison between GEP-NET G3 and GEP-NEC revealed no significant differences when all intensities of DLL3 expression were considered (8/15 DLL3-positive GEP-NET G3 vs. 58/133 DLL3-positive GEP-NEC; *p* = 0.508). However, strong DLL3 expression was predominantly observed in GEP-NEC, while GEP-NET G3 generally displayed lower DLL3 expression intensity, with only one case showing strong expression (1/15 strongly DLL3-positive GEP-NET G3 vs. 34/133 strongly DLL3-positive GEP-NEC; *p* = 0.003).

### DLL3 Expression in Primary NEN Vs. Metastases

In exploratory analyses of DLL3 expression between primary NEN and their corresponding metastases, we observed a high concordance. Overall DLL3 expression (positive vs. negative) was concordant in 92.5% of the paired samples (62/67, *p* < 0.001). When evaluating specific DLL3 expression groups, the identical IRS score was observed in 91% of the paired samples (61/67, *p* < 0.001). Among the six pairs with changes in DLL3 expression between the primary tumour and metastasis, an increase in DLL3 expression was observed in five cases, mostly from a DLL3 negative primary to a DLL3 positive metastasis. In contrast, a single case of pulmonary LCNEC exhibited a complete loss of DLL3 expression in its corresponding metastasis. For further details, see Supplementary Table 2 and Supplementary Fig. 3.

### Prognostic Implications of DLL3 Expression in Pulmonary and Extrapulmonary NEN

DLL3 expression (any degree) was associated with worse OS in the overall NEN cohort (*p* < 0.001), as expected due to the association of DLL3 expression with NEC. No statistical differences between the different DLL3 expression groups were noted (data not shown).

In separate statistical analyses, no association of DLL3 expression with survival was observed in NEC (*p* = 0.32; data not shown), which was also the case in separate analyses in pulmonary (*p* = 0.708) and GEP-NEC (*p* = 0.87) (Supplementary Fig. 4A–B). In univariable analyses, DLL3 positive pulmonary TC/AC (*p* = 0.005; median OS not reached) (Supplementary Fig. 4C) and GEP-NET (*p* = 0.018; median OS: 80 vs. 106 months for DLL3-positive vs. DLL3-negative GEP-NET) (Supplementary Fig. 4D) showed shorter OS. However, this association was not retained in multivariable analyses adjusting for pTNM stage, grade, sex, and age (*p* = n.s. for both pulmonary carcinoids and GEP-NET; data not shown).

### DLL3 Expression in Non-neuroendocrine Carcinomas

We observed the expression of DLL3 in a collective of primary resected 479 non-neuroendocrine carcinomas including colorectal, gastric, pancreatic, and pulmonary carcinomas. Expression of DLL3 was rare in non-neuroendocrine carcinomas (6/479, 1.3%) with two gastric adenocarcinomas showing a weak expression (2/69, 2.9%) and four positive pulmonary carcinomas—one weak (1.3%), three moderate (3.8%)—while a strong expression was never observed and all colorectal and pancreatic adenocarcinomas were entirely negative (Fig. 1J).

## Discussion

Delta-like ligand 3 (DLL3) has emerged as a therapeutic target in pulmonary SCNEC [7, 8]. DLL3-directed T-cell engagers have demonstrated promising antitumoural efficacy in patients with relapsed/refractory tumours [13] and other types of agents (ADC/CAR T-cells) are currently under (pre-) clinical evaluation [7]. While these recent findings have strengthened the position of DLL3 as a protein of interest for these hard-to-treat cancers, limited data preclude conclusions about DLL3’s potential therapeutic role in other types of pulmonary NEN, extrapulmonary NEN and also non-neuroendocrine carcinomas.

Our study systematically investigated DLL3 expression in a comprehensive cohort of 1294 NEN and 479 non-neuroendocrine carcinomas, revealing significant differences not only between NEC and NET in general, but also among their various histological subtypes, grades, and anatomic localisations.

The majority of NEC exhibited DLL3 expression, with the highest expression frequency in SCNEC, which mostly showed a strong and diffuse positivity for the protein. This high prevalence of DLL3 expression in SCNEC aligns with previous studies in pulmonary SCNEC [26–29], that highlighted the therapeutic potential of DLL3 for these tumours. Gastroenteropancreatic SCNEC displayed comparable DLL3 expression levels compared to their pulmonary counterparts, indicating a relatively consistent expression pattern for this morphologic variant of NEC, giving hope that DLL3-targeted therapies might have a similar efficacy in extrapulmonary SCNEC.

However, DLL3 expression was less pronounced in other subtypes of NEC. Compared to SCNEC, we detected significantly lower rates in LCNEC and MiNEN, not only regarding general expression, but also in terms of expression intensity. In contrast to SCNEC, DLL3 expression was also significantly different between pulmonary and extrapulmonary neoplasms, with pulmonary LCNEC and MiNEN showing a much higher rate of DLL3 expression compared to their gastroenteropancreatic counterparts. The landscape of DLL3 expression appears to be more heterogenous in LCNEC and MiNEN, which indicates that some, especially of gastroenteropancreatic origin, may respond less effectively to therapies targeting DLL3.

Our analyses also revealed considerable differences between pulmonary carcinoids and GEP-NET. The comparatively frequent expression of DLL3 in TC/AC also renders these well-differentiated pulmonary NEN as potential targets for DLL3 based therapies. In GEP-NET, however, DLL3 expression was comparably rare, which means that DLL3 may not be a therapeutic option for most of these neoplasms. These results also allow for important conclusions regarding the significance of DLL3 for the differential diagnostic distinction between well-differentiated NEN and NEC. In the lung, DLL3 appears to be of little value for this distinction, as a significant proportion of pulmonary carcinoids are DLL3 positive, with frequent cases showing at least a moderate or even a strong expression. In GEP-NEN, DLL3 expression was significantly enriched in GEP-NEC (*p* < 0.001), with 43.6% of NEC cases exhibiting overall DLL3 positivity. In contrast, only 5.1% of GEP-NET cases expressed DLL3, and strong expression was observed in just 0.5% of them. However, these findings should be interpreted with caution, as our study included a relatively small number of GEP-NET G3 cases (n = 15), which represent the most challenging group to distinguish from NEC. When considering all expression intensities, the frequency of DLL3 expression in GEP-NET G3 was comparable to that in GEP-NEC. Strong DLL3 expression was present in both groups but was more frequent in GEP-NEC. Given the limited sample size of GEP-NET G3 in our study, further research is required to better assess the diagnostic value of DLL3 in this context. However, our preliminary data suggest that DLL3 has only limited diagnostic utility for distinguishing GEP-NET G3 from GEP-NEC.

One additional clinical aspect we aimed to explore was the behaviour of DLL3 expression during metastatic progression, as many NEN present with metastatic disease either at diagnosis or during the course of the disease [1–3]. To investigate this, we analysed DLL3 expression patterns in 67 metastatic samples from our cohort and observed a high concordance in expression. In the few discordant cases, we predominantly observed an increase in DLL3 expression, with only one case of an initially strongly positive pulmonary LCNEC exhibiting a complete loss of DLL3 expression in its metastasis. These findings suggest that, in most cases, DLL3 expression in metastases closely reflects that of the primary tumour. Therefore, when no biopsy from the primary tumour is available, DLL3 assessment in metastases may provide a reliable estimation of its expression in the primary site. However, in rare cases, additional testing of metastatic sites may be beneficial, particularly during disease progression and in tumours with an initially negative primary.

We also explored the potential prognostic impact of DLL3 expression. DLL3 expression showed no prognostic significance in pulmonary or GEP-NEC, which is unsurprising given the inherently aggressive nature of NEC. Exploratory univariate analyses suggested shorter overall survival in patients with DLL3 positive pulmonary carcinoids and GEP-NET; however, this association was not retained in multivariate analyses adjusted for stage and grade. Nevertheless, the observed univariate association between DLL3 positivity and poorer survival in well-differentiated NEN is notable and warrants further investigation in larger cohorts.

Finally, we wanted to explore the expression landscape of DLL3 in non-neuroendocrine carcinomas, in which DLL3 expression was exceedingly rare (and if found, mostly weak), which argues that expression of DLL3 is largely restricted to NEN. Interestingly, separate analysis of DLL3 expression in the neuroendocrine and non-neuroendocrine components of MiNEN showed a mostly concordant general expression between both components, although usually weaker in the non-neuroendocrine component. This observation suggests that anti-DLL3 targeted therapies might also show some degree of efficacy against the non-neuroendocrine component in MiNEN and aligns with the theory of a common ancestry of the neuroendocrine and non-neuroendocrine components in MiNEN [30, 31].

Due to the large size of our cohort, the majority of our analyses was performed on TMA, which means that our investigations might not fully reflect possible intratumoural heterogeneity of DLL3, which has been described by previous studies [32]. However, comparison of whole slides from one complete TMA block showed an excellent concordance between the results from whole slides and the TMA, so that we believe that this is a rather minor limitation. Another limitation is the relatively small number of paired metastatic samples available for comparative analyses between primary NEN and their metastases, which should be investigated on a broader scale in future studies.

In conclusion, our study provides a comprehensive overview of the expression landscape of DLL3 in NET and NEC, highlighting significant differences between various histopathological subtypes and the primary tumour’s localisation. Our analyses suggest that DLL3-based therapies could be effective for a substantial fraction of NEC and pulmonary carcinoids. However, since GEP-NET and non-neuroendocrine carcinomas rarely express DLL3, it appears to be a less promising target for these neoplasms. Future clinical trials should also focus on evaluating DLL3-targeted therapies in GEP-NEC, pulmonary LCNEC as well as pulmonary carcinoids, and should also investigate the potential role of DLL3 expression as a biomarker to refine patient selection. Further research is also needed to explore alternative therapeutic strategies for DLL3 negative tumours and to better understand the molecular mechanisms underlying DLL3 expression across the different types of NEN.

## Supplementary Information

Below is the link to the electronic supplementary material.

Supplementary file1 DLL3 expression in neuroendocrine and non-neuroendocrine carcinoma component of MiNEN A-F: MiNEN composed of a SCNEC (blue arrow) combined with a squamous cell carcinoma component (black arrow) (HE, A, 2x) demonstrating a strong membranous CK5/6 expression in the squamous cell carcinoma component (B, 2x) and a strong cytoplasmatic synaptophysin expression in the SCNEC component (C, 2x). DLL3 is expressed in both components, albeit with different expression intensities. The SCNEC component shows strong DLL3 expression intensity in >50% of tumour cells (E, IRS 9, 20x). In the squamous cell carcinoma component DLL3 is strongly expressed in <10% of tumour cells (F, IRS 3, 20x). G-I: MiNEN composed of a LCNEC (blue asterix) combined with an adenocarcinoma component (black asterix) (HE, G, 20x) demonstrating a negative DLL3 expression in the neuroendocrine carcinoma (blue asterix) as well as in the nonneuroendocrine carcinoma component (black asterix) (IRS 0; H, 20x and I, 40x).HE, hematoxylin and eosin; DLL3, Delta-like-protein 3; CK, cytokeratin; IRS, immunoreactive score; MiNEN, mixed neuroendocrine-non-neuroendocrine neoplasm; LCNEC, large-cell neuroendocrine carcinoma; SCNEC, small-cell neuroendocrine carcinoma. (PNG 21263 KB)

Supplementary file2 Frequency of DLL3 expression groups in NEN according to their detailed anatomic sides A: Frequency of DLL3 expression groups according to the IRS in NEC (including MiNEN) for their detailed tumour localisations. B: Frequency of DLL3 expression groups according to the IRS in NET (including AC/TC) for their detailed tumour localisations. DLL3, Delta-like-protein 3; NEN, neuroendocrine neoplasm; NET, neuroendocrine tumour; NEC, neuroendocrine carcinoma; MiNEN, mixed neuroendocrine-non-neuroendocrine carcinoma; TC, typical carcinoid; AC, atypical carcinoid; GEJ, gastroesophageal junction. (PNG 188 KB)

Supplementary file3 Frequency of DLL3 expression groups in primary NEN vs metastatic sides A-B: Example of pulmonary LCNEC with concordant strong DLL3 expression intensity in >80% of tumour cells in primary tumour (A, IRS 12, 20x) as well as metastasis (B, IRS 12, 20x). C-D: Example of gastric MiNEN with a complete negative DLL3 expression (C, IRS 0, 20x) in the LCNEC component and up to strong DLL3 expression intensity in <10% of neuroendocrine differentiated tumour cells in corresponding metastasis (D, IRS 3, 20x). E-F: Example of ileum NET G1 with complete negative DLL3 expression in primary tumour side (E, IRS 0, 20x) and increase of DLL3 expression with up to strong expression intensity in >10% of tumour cells meaning a moderate DLL3 expression in corresponding metastasis (F, IRS 6, 20x). G: Pie chart representing changes of IRS expression groups in metastases compared to their corresponding primary tumours. H: Sankey diagram demonstrating detailed shifts of IRS expression groups between primary tumours and metastases. DLL3, Delta-like-protein 3; LCNEC, large cell neuroendocrine carcinoma; MiNEN, mixed neuroendocrine-nonneuroendocrine carcinoma; NET, neuroendocrine tumour. (PNG 13271 KB)

Supplementary file4 Survival analyses (log-rank test) in DLL3 expression groups A: Univariate overall-survival analysis of simplified DLL3 expression groups (DLL3-negative vs. DLL3-positive) in pulmonary NEC. B: Univariate overall-survival analysis of simplified DLL3 expression groups (DLL3-negative vs. DLL3-positive) in GEPNEC. C: Univariate overall-survival analysis of simplified DLL3 expression groups (DLL3-negative vs. DLL3-positive) in pulmonary AC/TC. D: Univariate overall-survival analysis of simplified DLL3 expression groups (DLL3-negative vs. DLL3-positive) in GEPNET. DLL3, Delta-like-protein 3; NET, neuroendocrine tumour; NEC, neuroendocrine carcinoma; TC, typical carcinoid; AC, atypical carcinoid; GEP, gastroenteropancreatic. (PNG 1494 KB)

Supplementary file5 Overview of the cohort and DLL3 expression in different NEN (corresponding to Figure 1). (XLSX 17 KB)

Supplementary file6 Overview of DLL3 expression in metastases and comparison to their DLL3 expression in primary tumour. (XLSX 15 KB)

## Data Availability

Tissue and data from this manuscript are stored at the Institute of Pathology, Philipps University Marburg und University Hospital Marburg, Marburg, Germany and are available from the corresponding author upon reasonable request.
