# Supplementary figures and images for: DLL3 Expression in Neuroendocrine Carcinomas and Neuroendocrine Tumours: Insights From a Multicentric Cohort of 1294 Pulmonary and Extrapulmonary Neuroendocrine Neoplasms
Source: Endocr Pathol. 2025 Mar 28;36(1):9. doi: 10.1007/s12022-025-09854-3 (PMC11953094; doi:10.1007/s12022-025-09854-3)

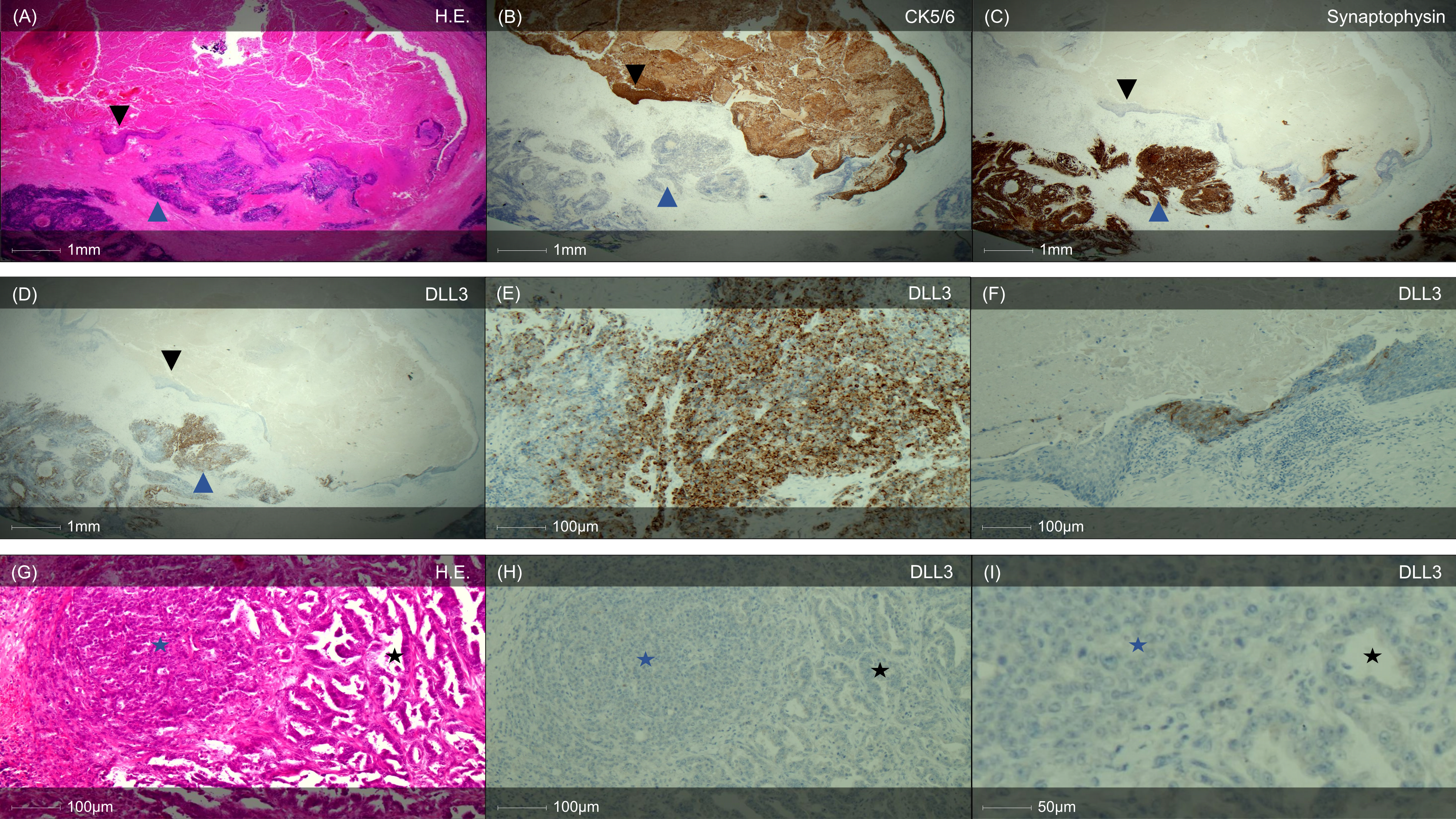

Supplement: Supplementary file 1 — Supplementary file1 DLL3 expression in neuroendocrine and non-neuroendocrine carcinoma component of MiNEN A-F: MiNEN composed of a SCNEC (blue arrow) combined with a squamous cell carcinoma component (black arrow) (HE, A, 2x) demonstrating a strong membranous CK5/6 expression in the squamous cell carcinoma component (B, 2x) and a strong cytoplasmatic synaptophysin expression in the SCNEC component (C, 2x). DLL3 is expressed in both components, albeit with different expression intensities. The SCNEC component shows strong DLL3 expression intensity in >50% of tumour cells (E, IRS 9, 20x). In the squamous cell carcinoma component DLL3 is strongly expressed in <10% of tumour cells (F, IRS 3, 20x). G-I: MiNEN composed of a LCNEC (blue asterix) combined with an adenocarcinoma component (black asterix) (HE, G, 20x) demonstrating a negative DLL3 expression in the neuroendocrine carcinoma (blue asterix) as well as in the nonneuroendocrine carcinoma component (black asterix) (IRS 0; H, 20x and I, 40x).HE, hematoxylin and eosin; DLL3, Delta-like-protein 3; CK, cytokeratin; IRS, immunoreactive score; MiNEN, mixed neuroendocrine-non-neuroendocrine neoplasm; LCNEC, large-cell neuroendocrine carcinoma; SCNEC, small-cell neuroendocrine carcinoma. (PNG 21263 KB) [file 12022_2025_9854_MOESM1_ESM.png]

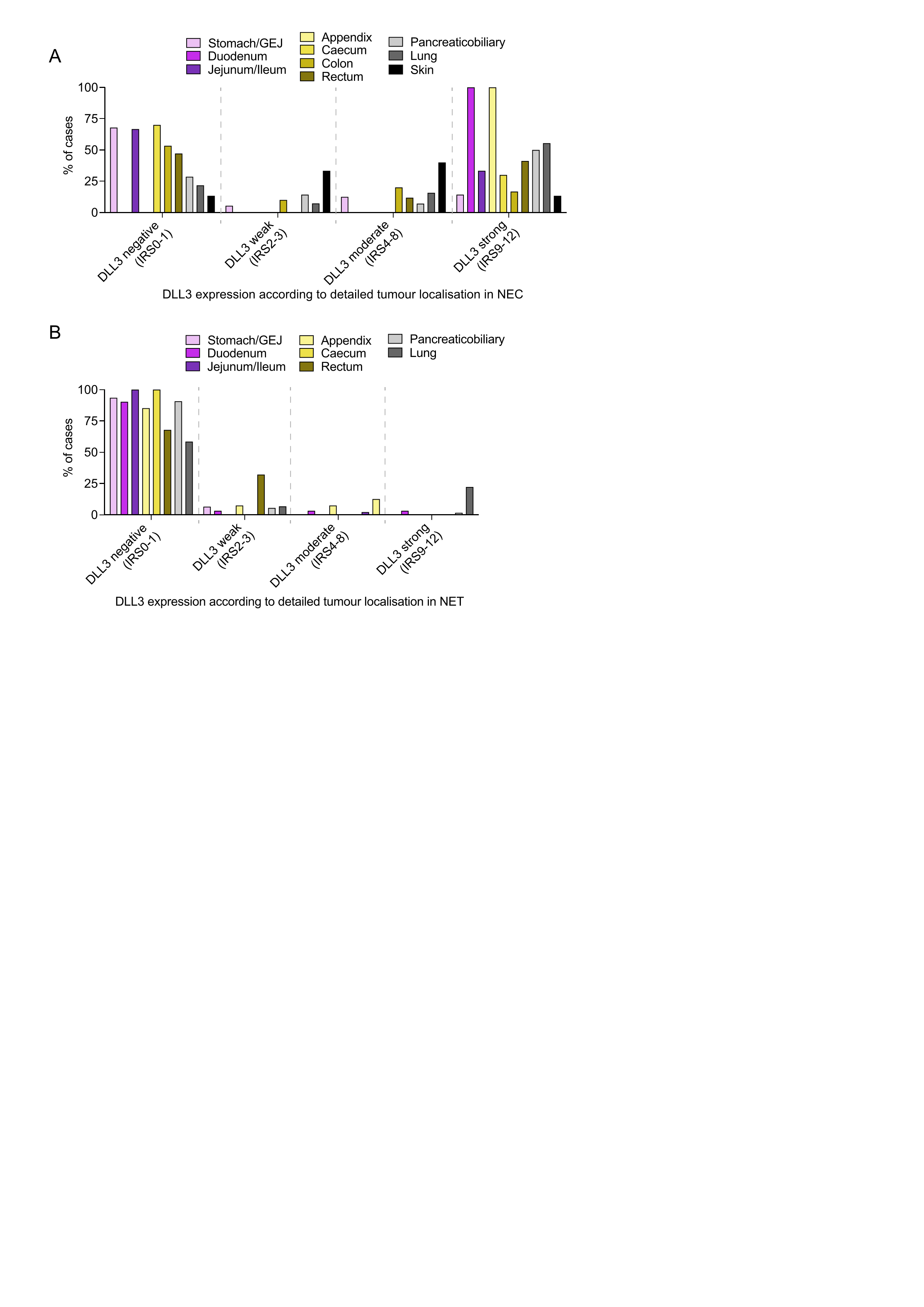

Supplement: Supplementary file 2 — Supplementary file2 Frequency of DLL3 expression groups in NEN according to their detailed anatomic sides A: Frequency of DLL3 expression groups according to the IRS in NEC (including MiNEN) for their detailed tumour localisations. B: Frequency of DLL3 expression groups according to the IRS in NET (including AC/TC) for their detailed tumour localisations. DLL3, Delta-like-protein 3; NEN, neuroendocrine neoplasm; NET, neuroendocrine tumour; NEC, neuroendocrine carcinoma; MiNEN, mixed neuroendocrine-non-neuroendocrine carcinoma; TC, typical carcinoid; AC, atypical carcinoid; GEJ, gastroesophageal junction. (PNG 188 KB) [file 12022_2025_9854_MOESM2_ESM.png]

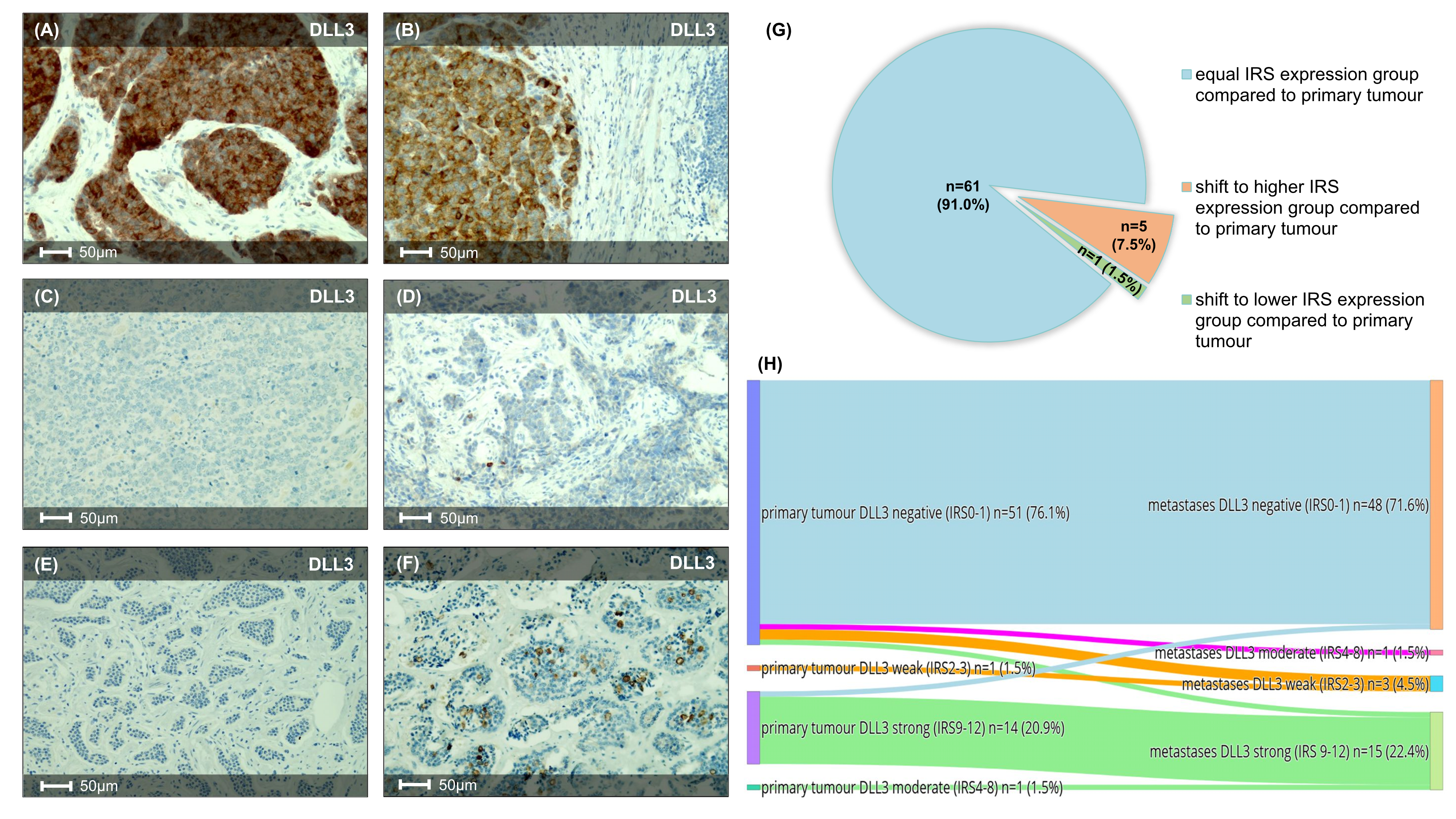

Supplement: Supplementary file 3 — Supplementary file3 Frequency of DLL3 expression groups in primary NEN vs metastatic sides A-B: Example of pulmonary LCNEC with concordant strong DLL3 expression intensity in >80% of tumour cells in primary tumour (A, IRS 12, 20x) as well as metastasis (B, IRS 12, 20x). C-D: Example of gastric MiNEN with a complete negative DLL3 expression (C, IRS 0, 20x) in the LCNEC component and up to strong DLL3 expression intensity in <10% of neuroendocrine differentiated tumour cells in corresponding metastasis (D, IRS 3, 20x). E-F: Example of ileum NET G1 with complete negative DLL3 expression in primary tumour side (E, IRS 0, 20x) and increase of DLL3 expression with up to strong expression intensity in >10% of tumour cells meaning a moderate DLL3 expression in corresponding metastasis (F, IRS 6, 20x). G: Pie chart representing changes of IRS expression groups in metastases compared to their corresponding primary tumours. H: Sankey diagram demonstrating detailed shifts of IRS expression groups between primary tumours and metastases. DLL3, Delta-like-protein 3; LCNEC, large cell neuroendocrine carcinoma; MiNEN, mixed neuroendocrine-nonneuroendocrine carcinoma; NET, neuroendocrine tumour. (PNG 13271 KB) [file 12022_2025_9854_MOESM3_ESM.png]

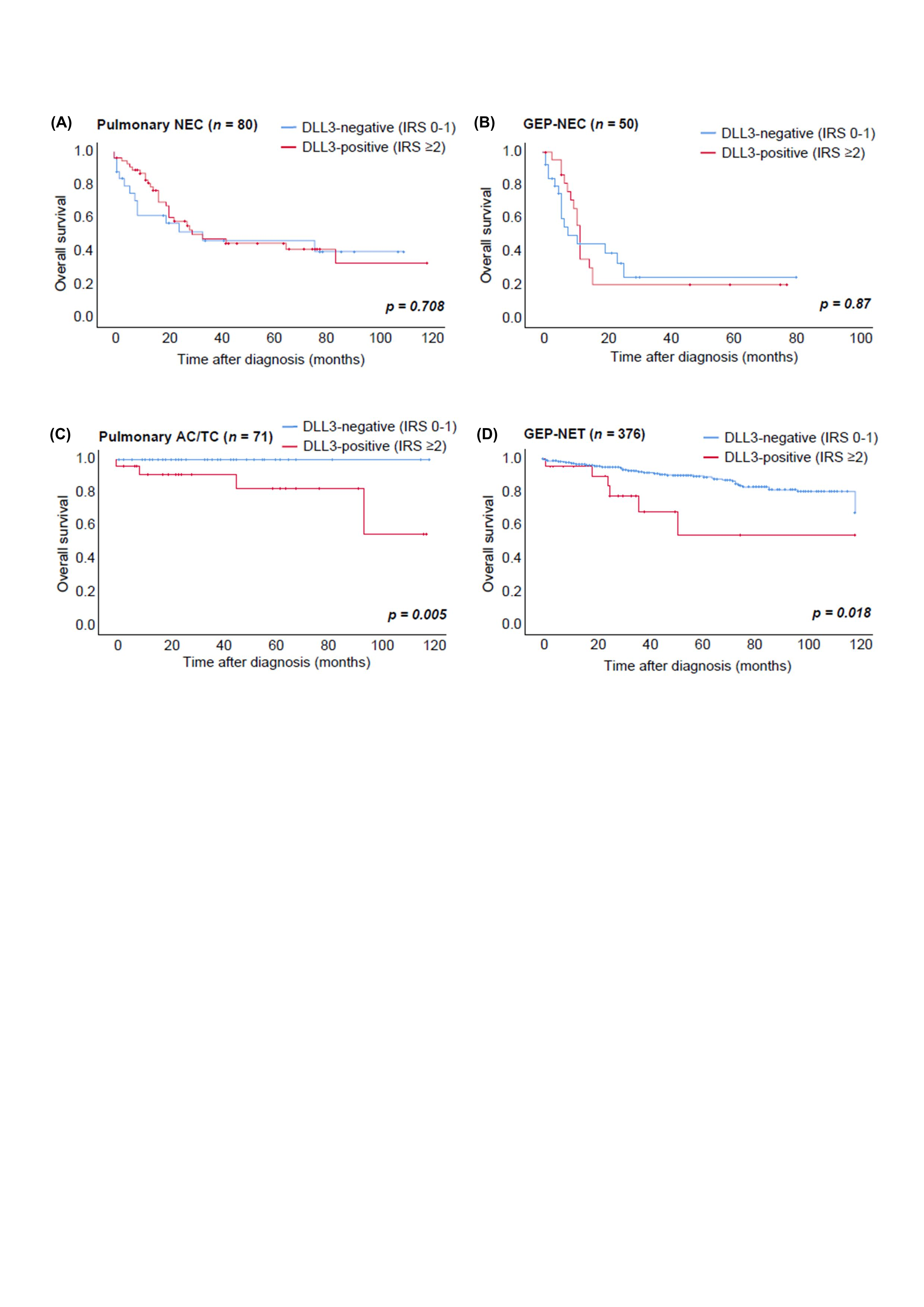

Supplement: Supplementary file 4 — Supplementary file4 Survival analyses (log-rank test) in DLL3 expression groups A: Univariate overall-survival analysis of simplified DLL3 expression groups (DLL3-negative vs. DLL3-positive) in pulmonary NEC. B: Univariate overall-survival analysis of simplified DLL3 expression groups (DLL3-negative vs. DLL3-positive) in GEPNEC. C: Univariate overall-survival analysis of simplified DLL3 expression groups (DLL3-negative vs. DLL3-positive) in pulmonary AC/TC. D: Univariate overall-survival analysis of simplified DLL3 expression groups (DLL3-negative vs. DLL3-positive) in GEPNET. DLL3, Delta-like-protein 3; NET, neuroendocrine tumour; NEC, neuroendocrine carcinoma; TC, typical carcinoid; AC, atypical carcinoid; GEP, gastroenteropancreatic. (PNG 1494 KB) [file 12022_2025_9854_MOESM4_ESM.png]
